# Mass spectrometry dataset for LC-MS metabolomics analysis of *Garcinia mangostana* L. seed development

**DOI:** 10.1016/j.dib.2018.10.025

**Published:** 2018-10-12

**Authors:** Othman Mazlan, Wan Mohd Aizat, Syarul Nataqain Baharum, Kamalrul Azlan Azizan, Normah Mohd Noor

**Affiliations:** Institute of Systems Biology (INBIOSIS), Universiti Kebangsaan Malaysia (UKM), 43600 UKM Bangi, Selangor, Malaysia

**Keywords:** Mangosteen, Seed development, LC-MS, Metabolomics, Metabolome

## Abstract

*Garcinia mangostana* L. (mangosteen) seed is recalcitrant, prone to low temperature and drying which limit its long-term storage. Therefore, it is imperative to understand the metabolic changes throughout its development, to shed some light into the recalcitrant nature of this seed. We performed metabolomics analysis on mangosteen seed at different stages of development; six, eight, ten, twelve and fourteen weeks after anthesis. Seed samples were subjected to methanol extraction prior analysis using liquid chromatography – mass spectrometry (LC-MS). The MS data acquired were analyzed using ProfileAnalysis (version 2.1). This data article refers to the article entitled “Metabolomics analysis of developing *Garcinia mangostana* seed reveals modulated levels of sugars, organic acids and phenylpropanoid compounds” (Mazlan et al., 2018) [1].

## Specifications table

**Table:** 

Subject area	Biology
More specific subject area	Metabolomics
Type of data	Table
How data was acquired	Mass spectrometry data were obtained using Dionex Ultimate 3000 UHPLC+ platform (Thermo Scientific) with MicroTOF-QIII hybrid quadrupole-time of flight MS system (Bruker, Germany) with ESI in positive ionization mode.
Data format	XLSX format
Experimental factors	Mangosteen seed development; six, eight, ten, twelve and fourteen weeks after anthesis.
Experimental features	Methanol extracts were analyzed using LC-MS system and processed using ProfileAnalysis (version 2.1).
Data source location	UKM Bangi, Malaysia (2.922662°E, 101.786690°N)
Data accessibility	Supplementary Table 1
Related research article	Mazlan, O., Aizat, W. M., Baharum, S. N., Azizan, K. A. and Noor, N. M. 2018. Metabolomics analysis of developing *Garcinia mangostana* seed reveals modulated levels of sugars, organic acids and phenylpropanoid compounds. Sci. Hortic. 233, 323–330.

## Value of the data

• Metabolite profile was acquired from LC-MS platform for mangosteen seed development at different stages of development (six, eight, ten, twelve and fourteen weeks after anthesis) for the first time.
• This allows for identification of metabolites and their temporal changes during mangosteen seed development.
• Metabolite changes and regulation in mangosteen seed development elucidated using metabolomics analysis may improve our understanding about mangosteen seed development.
• Additionally, this data can be analyzed in complementary with transcriptomics data [2] to illuminate the metabolic or biosynthetic pathways in mangosteen seed development.

## 1 Data

This dataset is analyzed data of five mangosteen seed developmental stages (six, eight, ten, twelve and fourteen weeks after anthesis) that were imported into ProfileAnalysis and then tabulated in MS Excel (XLSX format).

## 2 Experimental design, materials and methods

### 2.1 Plant materials

Mangosteen fruits were acquired from experimental plots of Universiti Kebangsaan Malaysia (UKM) (GPS coordinate: 2.922662°E, 101.786690°N) [3]. The trees were between the age of 15–20 years old [1]. Fruits were marked at anthesis and their development observed. Fruits were randomly picked from 10 to 15 trees, and seeds were sampled and pooled from at least six different fruits (biological replicates) at different stages of development: six, eight, ten, twelve and fourteen weeks after anthesis and kept at −80 °C until experiment.

### 2.2 Metabolite extraction

Pooled whole seeds were pulverized to fine powder and 0.1 g fresh weight were utilized for metabolite extraction per replicate [4]. Each stage of development requires three replicates. Samples were extracted in 1400 µL of methanol and incubated at 70 °C for 15 min. Extracts were briskly mixed with 1 volume of water and then spun at 2200 *g* using a centrifuge. The supernatant was dried for four to six hours under vacuum before kept in −80 °C storage.

### 2.3 Liquid chromatography-mass spectrometry (LC-MS) protocol

This analysis employed Dionex Ultimate 3000 UHPLC+ platform (Thermo Scientific) fitted with MicroTOF-QIII MS system (Bruker, Germany) with electrospray ion source (ESI) [5], [6]. The adjustments for LC platform and MS systems follow Mazlan et al. [1]. The LC platform was set with 1.0 µL injection volume, 60 °C column temperature and 0.3 mL/min rate of flow. The mobile phase comprised of 0.1% formic acid in water and acetonitrile. Elution runs for 35 min with the preset gradients as in Mazlan et al. [1]. MS system with positive ESI ionization mode obtained all spectra (scanned within 50–1000 *m/z* range) in centroid mode.

### 2.4 Mass spectrometry data handling

Raw data from MS were imported into ProfileAnalysis version 2.1 (Bruker, Germany). For bucketing, the parameters for Find Molecular Features follow Mamat et al. [6] with smoothing width: 2. Bucket was generated with the following settings: retention time range (0.1–30.00 min), mass range (50–1000 *m/z*), normalization (largest bucket value in analysis). Then the data were tabulated using Microsoft Excel 2016.

## Appendix A Supplementary material

Supplementary Table 1. List of retention time (*R*t) to *m/z* values for mangosteen seed (six, eight, ten, twelve and fourteen weeks after anthesis) analyzed on LC-MS platform. *R*t: *m/z*, retention time to *m/z* values; six (W06), eight (W08), ten (W10), twelve (W12) and fourteen (W14) weeks after anthesis. Replicates (R1, R2, R3).
